# In Situ Synthesis of ZnO Nanoparticles Using Soy Protein Isolate for Sustainable and Multifunctional Finishing of Hemp Fabrics

**DOI:** 10.3390/polym18010116

**Published:** 2025-12-31

**Authors:** Benjamas Klaykruayat, Penwisa Pisitsak, Pisutsaran Chitichotpanya, Ritthisak Klanthip

**Affiliations:** 1 Department of Materials and Textile Technology, Faculty of Science and Technology, Thammasat University, Pathum Thani 12121, Thailand; 2 Center of Excellence in Smart Materials, Energy, Biochemistry, Food Technology, and Textile Innovation for Sustainable Environment, Thammasat University, Pathum Thani 12121, Thailand

**Keywords:** hemp fabric, infrared-assisted synthesis, soy protein isolate, sustainable textile finishing, UV protection, ZnO nanoparticles, antibacterial properties

## Abstract

This study presents an environmentally sustainable finishing approach for hemp fabrics by combining soy protein isolate (SPI) pretreatment with an in situ infrared (IR)-assisted synthesis of zinc oxide nanoparticles (ZnO NPs). IR heating was employed to reduce energy consumption while promoting efficient nanoparticle formation compared to conventional thermal processing, while SPI acted as a bio-based stabilizer to enable uniform ZnO NP distribution on the fabric surface. Transmission electron microscopy revealed predominantly spherical to polyhedral ZnO NPs with minimal agglomeration, and X-ray diffraction confirmed their characteristic wurtzite crystalline structure. Scanning electron microscopy coupled with energy-dispersive X-ray spectroscopy mapping further verified the homogeneous deposition of ZnO NPs on hemp fibers. The treated fabrics exhibited multifunctional performance, showing significantly enhanced ultraviolet (UV) protection with a UV protection factor (UPF) of 50+ compared with untreated hemp. Antibacterial activity against *Staphylococcus aureus* and *Escherichia coli* was confirmed by the AATCC TM147 test, while a quantitative AATCC TM100 assessment demonstrated an excellent antibacterial efficiency of 99.99% bacterial reduction against *S. aureus*. Additionally, the incorporation of 2 wt% SPI significantly improved fabric hydrophilicity and wettability. Overall, this work demonstrates a green and effective strategy for producing antibacterial and UV-protective hemp textiles.

## 1. Introduction

The growing demand for multifunctional textile materials that integrate protection, hygiene, and comfort has driven significant advances in sustainable finishing technologies for natural fibers. Among these, hemp (*Cannabis sativa*) has emerged as a promising substance owing to its outstanding mechanical strength, rapid renewability, low environmental footprint, and inherent ultraviolet (UV)-shielding properties [1]. However, untreated hemp fabrics inherently lack the functional properties required for advanced applications such as outdoor apparel and hygienic textiles, especially regarding UV protection factor (UPF), antibacterial efficacy, and wash durability [2].

Surface modification of nanoparticles—particularly zinc oxide (ZnO) nanoparticles (NPs)—has emerged as a promising approach to impart UV shielding and antibacterial functionalities to textile materials. ZnO NPs exhibit a wide band gap (~3.37 eV), enabling strong UV absorption, while their reactive surface sites promote the generation of reactive oxygen species (ROS) and the release of Zn^2+^ ions, which together contribute to potent bactericidal activity against Gram-positive and Gram-negative microorganisms [3,4]. A study investigated ZnO NPs on cotton, polyester, and blended fabrics to achieve UV protection and antimicrobial functionality [5]. However, the deposition of ZnO on natural fibers continues to pose significant challenges, including NP aggregation, poor adhesion, compromised moisture permeability, and limited wash durability.

To address these limitations, two strategies have shown promise: (i) biopolymer pretreatment usage—including proteins and polysaccharides—to enhance NP adhesion and dispersion on fiber surfaces, and (ii) novel energy-assisted synthesis techniques—including ultrasonication, microwave, and infrared (IR)—to enable in situ NP formation with controlled morphology. For instance, protein-functionalized surfaces can act as nucleation templates for NP, thus minimizing agglomeration and enhancing wash durability [6]. Similarly, IR heating accelerates nucleation kinetics, reduces processing time, and promotes uniform NP growth directly on textile substrates [7].

This study aims to develop a facile, rapid, and environmentally sustainable finishing process for hemp fabrics via integrating soy protein isolate (SPI) pretreatment with in situ IR-assisted ZnO NP synthesis. SPI—a renewable, biodegradable, and low-cost biopolymer derived from soybeans—acts as a bio-stabilizer and an interfacial adhesion promoter during ZnO NP formation. The IR-assisted approach enables direct in situ synthesis of ZnO NPs on the fabric surface at moderate temperature (60 °C, 60 min), significantly reducing processing time and energy consumption compared to that of the conventional thermal method. Moreover, compared with microwave or ultrasound treatments, IR heating offers better compatibility with continuous and scalable textile processing and reduces the risk of fiber damage. The treated fabrics were comprehensively evaluated in terms of flexural rigidity, tensile properties, surface wettability, ultraviolet protection factor (UPF), and antibacterial performance against *Escherichia coli* and *Staphylococcus aureus.*

## 2. Materials and Methods

### 2.1. Materials

Plain-woven hemp fabrics (198 g/m^2^) were purchased from a local supplier in Thailand. Zinc nitrate hexahydrate (Zn (NO_3_)_2_·6H_2_O) was obtained from QReC™, Auckland, New Zealand. Ammonium hydroxide (NH_4_OH, 1 M) was obtained from Loba Chemie, Bombay, India. Food-grade SPI and a commercial SAC emulsion binder (Appretan N96131, SAC, Värnamo, Sweden) were supplied by Vickers Laboratories Ltd., Pudsey, UK. Double-distilled water was used in all experimental procedures.

### 2.2. Methods

#### 2.2.1. Preparation of Soy Protein Isolate-Coated Hemp Fabric

Hemp fabrics were cut into specimens (24 × 15 cm) and immersed for 10 min in SPI solutions at varying concentrations (0.5, 1.0, 1.5, 2.0, and 3.0% *w*/*v*). The samples were padded using a laboratory padder (LABTEC, Model P-AO, Pingzhen City, Taiwan) at ambient temperature and subsequently dried in a mini-stenter at 100 °C for 3 min.

#### 2.2.2. Infrared-Assisted In Situ Synthesis of ZnO Nanoparticles

SPI-pretreated hemp fabrics were immersed in a 0.2 M zinc nitrate solution (40 mL per sample) for 5 min. Subsequently, 40 mL of 1 M ammonium hydroxide was added dropwise to initiate the in situ formation of Zn (OH)_2_, which was subsequently dehydrated to ZnO during heating. The fabrics were then transferred to an IR-assisted dyeing apparatus and processed at 60 °C for 60 min. After treatment, samples were air-dried, soaped at 60 °C for 15 min, rinsed twice with deionized water, and air-dried again to remove loosely bound NPs.

#### 2.2.3. Binder Treatment

ZnO-loaded fabrics were immersed in a 3% (*w*/*v*) SAC solution, padded to achieve a wet pick-up of approximately 80%, and dried in a mini-stenter (LABTEC, Pingzhen City, Taiwan) at 100 °C for 3 min. The treated samples were subsequently cured at 150 °C for 5 min.

#### 2.2.4. Characterization

##### X-Ray Diffraction

The crystalline structure of ZnO NPs and treated fabrics was analyzed using X-ray diffraction (XRD) on a Bruker AXS Model D8 Advance diffractometer (Bruker AXS GmbH, Karlsruhe, Germany). Measurements were conducted with a Cu radiation source (λ = 1.5406 Å) operated at a voltage of 40 kV and a current of 40 mA, over a 2θ range of 5–80°, at a scanning rate of 0.20° per s.

##### Transmission Electron Microscopy Analysis

The morphology of ZnO NPs deposited on the treated fabrics was examined using transmission electron microscopy (TEM, JEM-2100, JEOL, Tokyo, Japan). For sample preparation, small pieces of ZnO-coated fabric were immersed in deionized water and ultrasonicated to release the NPs into suspension. A drop of the resulting dispersion was placed onto a carbon-coated copper grid and air-dried prior to imaging. TEM analysis was conducted at an accelerating voltage of 80 kV to determine particle size and morphology.

##### Scanning Electron Microscope and Energy-Dispersive X-Ray Spectroscopy Analysis

The surface morphology of the fabrics was examined using a scanning electron microscope (SEM, JEOL JSM-5410LV, Tokyo, Japan). The samples were sputter-coated with a thin gold/palladium layer, and micrographs were captured at an accelerating voltage of 5 kV. Additionally, a field-emission SEM (JEOL JSM-7800F, Tokyo, Japan) equipped with energy-dispersive X-ray spectroscopy (EDS) was employed to analyze the elemental composition and distribution on the fiber surfaces.

##### Fabric Stiffness Analysis

The flexural rigidity of the fabrics was measured according to British Standard 3356 [8] using a fixed-angle flexometer (TABER^®^ Industries, New York, NY, USA). Fabric specimens (2.5 × 20 cm) were placed on the instrument platform and allowed to bend under their own weight until reaching the standard reference angle, at which point the bending length (C) was recorded. The flexural rigidity (G) was then calculated using Equation (1):

G = 0.1 × *M* × *C*^3^ (1)

where G represents the flexural rigidity (mg·cm), M is the fabric weight per unit area (mg/cm^2^), and C denotes the bending length (cm). The constant 0.1 is used to convert the units appropriately according to the standard calculation method for fabric stiffness.

##### Mechanical Properties: Tensile Strength and Elongation at Break

The tensile properties of the fabrics were measured using a universal testing machine equipped with a 5-kN load cell (ZwickRoell GmbH, Ulm, Germany)) following ASTM D5035-95 (Strip Method) [9], with a gauge length of 75 mm and testing speed of 300 mm/min. The tensile strength and elongation at break were recorded to assess the mechanical performance of the untreated and treated fabrics.

##### Wettability Analysis: Contact Angle Measurement

The water contact angle of the fabrics was measured using the sessile drop technique with a Theta Lite tensiometer (Theta Lite TL100, Biolin Scientific, Gothenburg, Sweden). A 10 μL droplet of deionized water was dispensed from a micro syringe positioned approximately 2 mm above the fabric surface, and its profile was captured at 6 fps. The contact angle was automatically determined using OneAttension software (Biolin Scientific AB, Gothenburg, Sweden; software included with the instrument). The reported values represent the average of five measurements, taken within the first 3 s after the droplet contacted the sample.

##### Ultraviolet Protection Factor (UPF)

The UV protection of the fabric was evaluated using AATCC Test Method 183-2010 [10], which measures transmittance within the 280–400 nm wavelength range with a double-beam scanning spectrophotometer (Camspec M550SPF, Spectronic Camspec Ltd., Leeds, UK). The UPF, as well as the percentages of UVA and UVB blocking, was reported.

##### Wash Fastness

The wash fastness of the treated fabrics (Hemp + ZnO and Hemp + 2% Soy + ZnO) was evaluated using a single washing cycle according to ISO 6330:2012 [11]. The test was conducted in a front-loading washing machine at 40 ± 2 °C with a standard detergent (liquor ratio 1:50). The cycle included optimal agitation, rinsing, and air drying at room temperature, without the use of softeners or optical brighteners.

##### Antibacterial Activity

The antibacterial efficacy was initially evaluated according to AATCC TM147 [12] using *Staphylococcus aureus* (ATCC 6538) [13] and *Escherichia coli* (ATCC 8739) [14]. The fabric specimens were placed in direct contact with bacterial streaks on nutrient agar plates and incubated at 37 °C for 24 h. The width of the inhibition zone surrounding each specimen was recorded. To quantitatively assess antibacterial performance, the antibacterial activity against *S. aureus* was additionally determined following AATCC TM100 [15]. Briefly, fabric samples were inoculated with a known concentration of bacterial suspension and incubated at 37 °C for 24 h. After incubation, bacteria were eluted from the specimens, serially diluted, and plated on nutrient agar. The number of viable bacteria was counted, and the antibacterial efficiency was expressed as the percentage reduction in bacterial population relative to the untreated control.

## 3. Results and Discussion

### 3.1. X-Ray Diffraction Analysis

**Figure 1:**
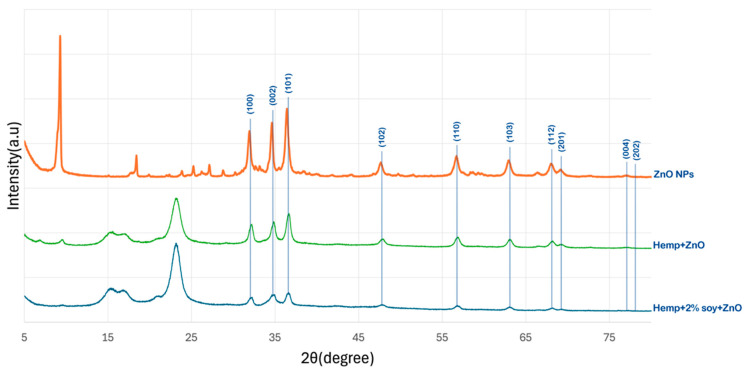
XRD patterns of ZnO NPs, hemp fabrics treated with ZnO NPs, and hemp fabric pretreated with 2% SPI followed by in situ synthesis of ZnO NPs.

Figure 1 presents the XRD patterns of pristine hemp fabric, ZnO-NPs, ZnO-coated hemp fabric, and hemp fabric pretreated with 2% SPI followed by in situ synthesis of ZnO-NPs. Distinct diffraction peaks at 2θ values of approximately 31.9°, 34.5°, 36.4°, 47.6°, 56.7°, 62.9°, 66.5°, 68.0°, 72.6°, and 76.8°, corresponding to the (100), (002), (101), (102), (110), (103), (200), (112), (004), and (202) crystal planes of the hexagonal wurtzite ZnO structure (JCPDS No. 36-1451), respectively, confirm the formation of crystalline ZnO NPs, consistent with previous reports [16,17,18,19].

For the hemp fabric pretreated with SPI, the ZnO-related diffraction peaks remained observable but exhibited noticeably reduced intensity compared to those of the ZnO-NP–finished fabrics without SPI. This behavior indicated that SPI acted as a bio-stabilizing agent that suppressed ZnO crystal growth by forming a proteinaceous coating layer, leading to smaller crystallite sizes and increased structural disorder. Proteins are known to function as bio-stabilizers and nucleating agents by coordinating Zn^2+^ ions through amine and carboxyl functional groups, thereby promoting controlled nucleation, more uniform crystallite formation, and effective anchoring of ZnO on the fiber surface [20].

In addition to restricting crystallite growth, the presence of the SPI layer may partially attenuate the ZnO diffraction signal by increasing background scattering and reducing the effective diffracting volume of ZnO. Consequently, the reduced XRD peak intensity can be attributed to the combined effects of diminished ZnO crystallite size and signal attenuation caused by the overlying SPI coating layer. Similar reductions in ZnO XRD peak intensity have been reported in previous studies where ZnO nanoparticles were surface-modified or coated with organic materials, such as plant extracts or poly (vinyl alcohol), which either limit crystal growth or partially mask the diffraction signal of ZnO nanocrystals [21,22].

A low-angle diffraction peak at 2θ ≈ 9° was observed in the XRD pattern of the ZnO-NPs sample obtained from the bulk reaction medium. This peak can be attributed to a zinc hydroxide-related intermediate phase, such as layered zinc hydroxide or Zn (OH)_2_/hydroxy salt phases, which commonly form during the early stages of ZnO nucleation under alkaline conditions [23,24]. During synthesis, the addition of ammonium hydroxide to the zinc nitrate solution resulted in the formation of white precipitates in the bulk solution, which were collected separately and further analyzed by XRD. In contrast, this low-angle peak was not detected in the XRD patterns of the fabric-treated samples, regardless of SPI pretreatment. This observation indicates that the hydroxide intermediate did not persist on the textile substrate and was subsequently transformed into crystalline ZnO during IR heating. The presence of the fiber surface, with or without SPI, promotes heterogeneous nucleation and interfacial interactions, favoring the direct formation of wurtzite ZnO on the fabric and suppressing the retention of layered hydroxide phases. Consequently, only the characteristic diffraction peaks of wurtzite ZnO are observed in the fabric-treated samples, consistent with previous reports on ZnO formation from hydroxide intermediates [25].

### 3.2. Transmission Electron Microscopy (TEM) Analysis

**Figure 2:**
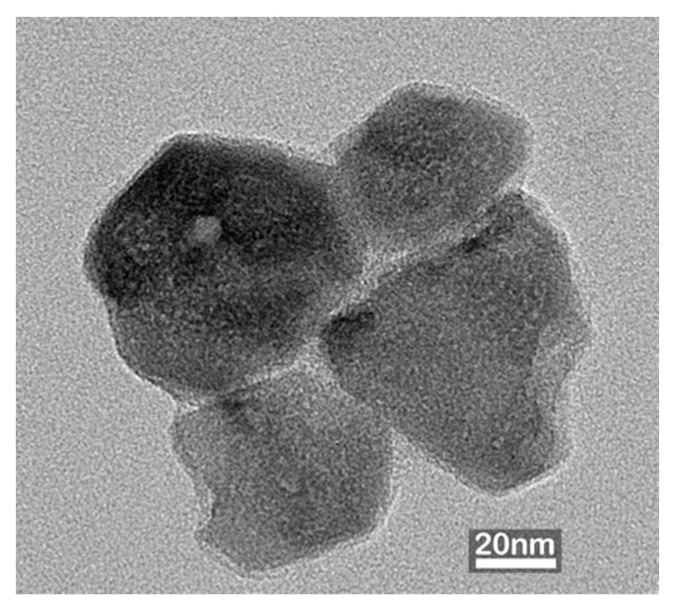
TEM images of ZnO nanoparticles synthesized through the in situ IR-assisted process, showing spherical to polyhedral morphology with slight agglomeration.

Figure 2 shows the TEM micrographs of ZnO NP synthesized through the in situ IR-assisted method. The NPs exhibited predominantly spherical-to-polyhedral morphologies with slight agglomeration, typical of ZnO formed under aqueous conditions due to surface energy-driven interactions among primary crystallites [16,17]. The particle size distribution of the synthesized ZnO NPs exhibited an average particle diameter of 57.0 ± 12.6 nm, consistent with the nanoscale crystallite sizes reported for hydrothermally or chemically synthesized ZnO NPs in previous studies [18,19,20].

### 3.3. Scanning Electron Microscope and Energy-Dispersive X-Ray Spectroscopy Analysis

Table 1 presents the SEM micrographs of untreated hemp fabric and ZnO NP-functionalized hemp fabrics under different finishing conditions. The pristine hemp fibers showed smooth surfaces with visible natural grooves typical of cellulose-based bast fibers. After ZnO deposition, the fiber surface became noticeably rougher, indicating successful NP adhesion. In the SPI-pretreated sample, ZnO deposits were more uniformly distributed, suggesting that SPI promotes NP nucleation and binding through interactions with Zn^2+^ ions and cellulose functional groups [16,18]. The binder-added formulation exhibited greater surface coverage, possibly due to the film-forming nature of the styrene–acrylic binder, which embeds ZnO NPs more firmly into the fiber matrix.

### 3.4. Fabric Stiffness Analysis

**Figure 3:**
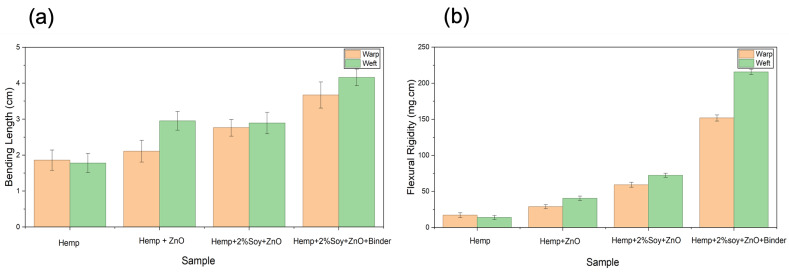
(**a**) Bending length of untreated and treated hemp fabrics in the warp and weft directions; (**b**) flexural rigidity of untreated and treated hemp fabrics in the warp and weft directions.

The stiffness of hemp fabrics before and after antibacterial finishing was evaluated through bending length and flexural rigidity analysis using Equation (1). The flexural rigidity of hemp fabrics significantly increased after ZnO deposition in warp and weft directions compared to that of the untreated hemp fabric (Table 2 and Figure 3). This increase is due to inorganic NP adhesion, which fills fiber interstices and enhances the structural consolidation of the yarns [16,17]. The addition of SPI and styrene–acrylic binder further increased the flexural rigidity of the fabrics. SPI acted as a biopolymeric adhesive that improved NP anchorage while preserving fiber flexibility and preventing excessive rigidity [18]. The styrene–acrylic binder produced a continuous film that bound ZnO NPs to the fiber surface, bridged adjacent fibers, and thus decreased fiber mobility [20]. The fabric treated with ZnO, SPI, and styrene–acrylic binder exhibited the highest increase in flexural rigidity—approximately 786% (warp) and 1443% (weft)—compared to that of the untreated hemp. Although this increase in stiffness affects the tactile sensation of the fabric, leading to a stiffer hand feel and reduced drape, the treated fabrics in this study are primarily intended for technical applications, such as protective, antibacterial, or UV-protective materials, where dimensional stability, structural integrity, and durability are prioritized over softness.

### 3.5. Mechanical Properties: Tensile Strength and Elongation at Break

**Figure 4:**
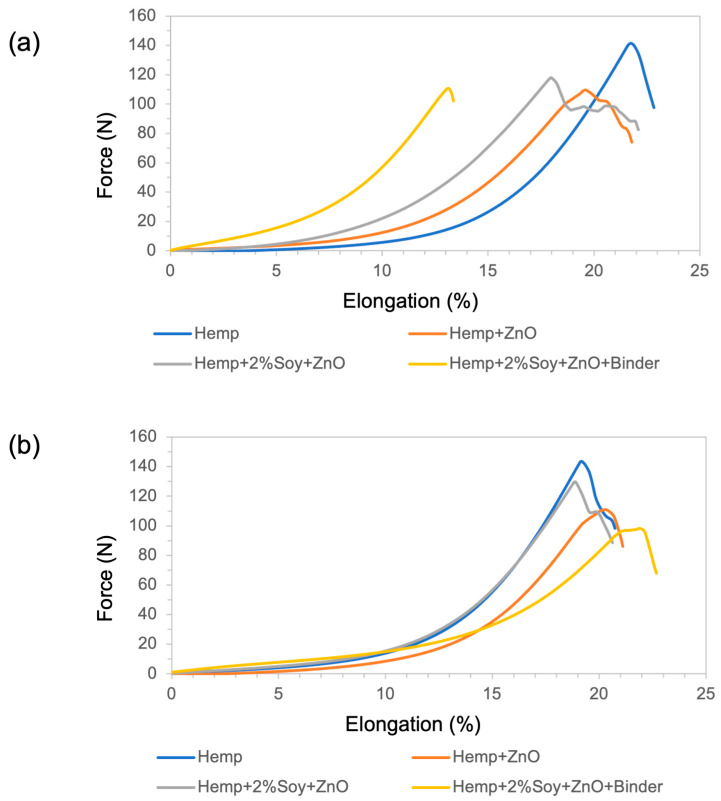
(**a**) Force–elongation behavior in the warp direction and (**b**) force–elongation behavior in the weft direction of untreated and ZnO-functionalized hemp fabrics with and without soy protein and binder.

Tensile properties were evaluated to investigate the influence of ZnO functionalization and polymeric additives on the mechanical performance of hemp fabrics, following ASTM D5035-95. The tensile strength and elongation at break in both the warp and weft directions are summarized in Table 3. Representative tensile force–elongation curves of the untreated and treated hemp fabrics are presented in Figure 4. The results are summarized in Table 3.

The untreated hemp fabric showed the highest tensile strength due to its native cellulose fibrillar structure and intact hydrogen-bonding network [16,26]. After ZnO NP deposition, tensile strength decreased in warp and weft directions, owing to increased brittleness resulting from the inorganic particle embedding that disrupted fiber mobility [20]. The hemp + 2%soy + ZnO exhibited the highest tensile strength compared to that of the other treated samples, suggesting that SPI reduces ZnO NPs agglomeration. The smaller size and better dispersion of ZnO NPs enhanced tensile properties compared to the fabric treated with ZnO. Moreover, the SPI coating was compatible with the hemp fibers and reinforced the coated fabric. In contrast, the rigid SAC reduced fiber flexibility, creating stress concentration sites that led to premature fracture under tensile loading [17].

Regarding elongation behavior, treated fabrics exhibited slightly reduced or comparable elongation at break relative to the untreated hemp, indicating moderate stiffening induced by the inorganic and polymeric finishing components [16,20]. This trend is consistent with the flexural rigidity results, where increased bending length reflects higher fabric stiffness and restricted yarn and fiber mobility, which typically leads to reduced fabric extensibility according to textile mechanics principles [27].

### 3.6. Wettability Analysis: Contact Angle Measurement

**Figure 5:**
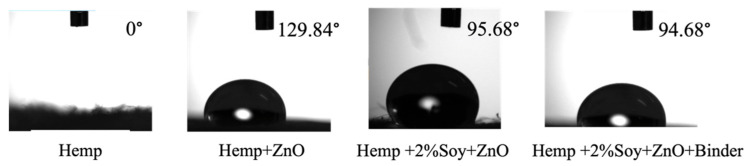
Initial water contact angles of untreated and finished hemp fabrics with ZnO, SPI, and binder treatments.

Figure 5 shows the results of static water contact angle measurements. The pristine hemp fabric showed a 0° contact angle, indicating immediate water absorption due to the hydrophilic nature of cellulose [28,29]. After ZnO NP finishing, the contact angle significantly increased to 129.84°, indicating that the surface became hydrophobic due to surface roughening caused by the presence of ZnO NPs. However, SPI pretreatment reduced the contact angle to 94.68°, suggesting that its hydrophilic functional groups (-NH_2_, -COOH) partially counteract the hydrophobicity induced via ZnO NPs [18]. Therefore, SPI enhances comfort performance, including sweat transfer, in next-to-skin textile applications. The SAC binder had little effect on the contact angle value of the coated fabric (95.68°).

### 3.7. Ultraviolet Protection Factor

The ultraviolet (UV) protection performance of hemp fabrics before and after functional finishing was evaluated using the ultraviolet protection factor (UPF) rating system, which reflects the effectiveness of a textile in blocking ultraviolet radiation in both the UVA (315–400 nm) and UVB (280–315 nm) regions. As summarized in Table 4, a pronounced enhancement in UV-blocking efficiency was observed after ZnO nanoparticle (NP) deposition.

Pristine hemp exhibited UPF 21.1, providing good UV protection, due to natural absorbers in hemp fibers, including pigments and lignin [30,31]. Incorporating ZnO NPs, SPI, and SAC improved UV protection. Fabrics treated with only ZnO achieved a 32.5 UPF rating, classified as a very good level. This is attributed to the intrinsic wide-bandgap semiconductor properties of ZnO, which efficiently absorb and scatter ultraviolet A and ultraviolet B photons [19,20,32]. The UV-absorbing properties of ZnO NPs have been well documented [33,34,35,36]. Incorporating SPI or SAC increased the UPF rating to over 50, achieving an excellent level and demonstrating the effectiveness of these polymeric additives in blocking UV radiation. This is expected as SPI and SAC contained UV-absorbing moieties including peptide bonds in SPI and aromatic rings in both additives [37,38]. Also, the polymeric coatings increased the effective fabric thickness and compactness, leading to enhanced light scattering and a longer optical path for UV radiation within the fabric structure. These combined effects significantly reduce UVA and UVB transmission, thereby explaining the high UPF values observed.

### 3.8. Antibacterial Activity

The antibacterial activity of the functionalized hemp fabrics was qualitatively evaluated using the agar diffusion assay according to AATCC 147 (zone of inhibition method). Table 5 and Table 6 show the results for *Escherichia coli* (Gram-negative) and *Staphylococcus aureus* (Gram-positive). No inhibition zone was observed for untreated hemp fabric, indicating the absence of inherent antibacterial activity. In contrast, fabrics treated with ZnO NPs exhibited clear bacterial growth inhibition, as ZnO generates ROS, disrupts bacterial membranes, and destabilizes protein and DNA structures [4,27,39]. Samples treated with ZnO NPs combined with SPI exhibited distinct antibacterial activity, showing similar zones of inhibition, compared to those treated with ZnO NPs alone. All treated fabrics showed a reduction in inhibition zone diameter after laundering, indicating partial loss of ZnO due to mechanical washing stress. The SPI pretreatment neither diminished the antibacterial activity nor adversely affected its durability. In addition to preserving antibacterial performance, SPI pretreatment contributed to improvements in tensile strength, surface wettability, and ultraviolet protection. By contrast, the use of SAC binder adversely affected these physical properties, particularly by increasing fabric stiffness. Consequently, SAC-treated samples were not further evaluated for antibacterial efficacy.

The antibacterial performance of the treated hemp fabrics was further quantitatively evaluated using the AATCC TM100 test method, and the corresponding results and representative test images are presented in Table 7. The percentage reduction in viable bacteria was calculated as the average of three independent measurements. Fabrics treated with either ZnO NPs alone or ZnO NPs in combination with SPI exhibited identical antibacterial efficiencies of 99.99% against *S. aureus.*

## 4. Conclusions

This study successfully developed a novel antibacterial finishing process for hemp fabrics by combining SPI pretreatment with an IR-assisted in situ synthesis of ZnO NPs. IR heating was used as an energy-efficient method to convert zinc nitrate to ZnO NPs. SPI pretreatment on the fabric generated active binding sites for subsequent ZnO nucleation and acted as a bio-stabilizer. SAC was used as a binder. The synthesized ZnO NPs were 57.0 ± 12.6 nm in diameter with spherical and polyhedral morphologies. The fabric stiffness increased progressively following treatment with ZnO NPs, SPI, and SAC. The tensile strength values decreased significantly in the presence of SAC binder or ZnO NPs, whereas SPI reinforced the ZnO-treated fabric. Incorporating ZnO NPs into hemp fabrics markedly increased the hydrophobicity of the fabric, as indicated by a contact angle of 129.84° compared to the untreated hemp, which absorbed the water droplet immediately. SPI pretreatment and the SAC binder slightly decreased hydrophobicity with contact angle values ranging from 94.68–95.68°. Although mechanical properties decreased, the treated fabrics exhibited multifunctional properties including antibacterial activity against *S. aureus* and *E. coli*, and excellent UV-shielding capability (UPF 50+), which can withstand washing. The SAC binder did not significantly enhance any fabric performance.

Although hemp fabric was selected as a model substrate in this study due to its natural abundance and cellulosic nature, the proposed finishing strategy is not limited to hemp alone. The underlying concept—SPI pretreatment combined with IR-assisted in situ ZnO nanoparticle synthesis—can be readily extended to other cellulosic and natural fiber–based textiles, such as cotton, linen, and regenerated cellulose fabrics. Consequently, this work provides a generalized and scalable framework for the development of eco-friendly antibacterial and UV-protective textile finishes.

## Figures and Tables

**Table 1:** SEM and EDS micrographs of (**a**) pristine hemp, (**b**) Hemp + ZnO, (**c**) Hemp + 2%Soy + ZnO, and (**d**) Hemp + 2%Soy + ZnO + Binder, demonstrating changes in surface morphology after NP deposition. Zn is represented by blue dots, C by red dots, and O by green dots.

Sample	Zn (%wt)	SEM	EDS
(**a**)			
Hemp	0	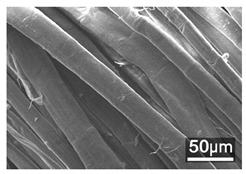	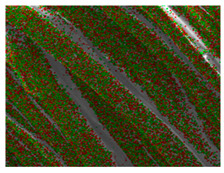
(**b**)			
Hemp + ZnO	30.12	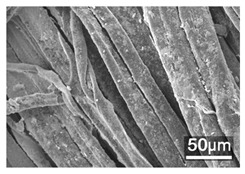	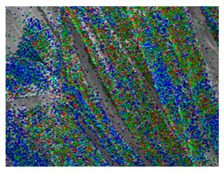
(**c**)			
Hemp + 2%Soy + ZnO	50.64	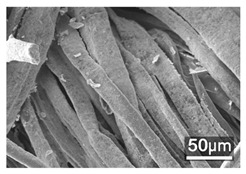	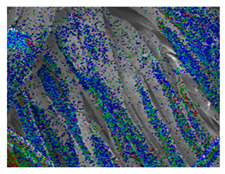
(**d**)			
Hemp + 2%Soy + ZnO + Binder	31.71	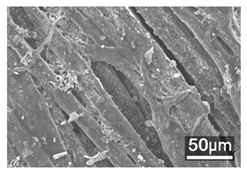	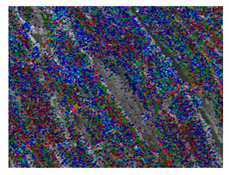

**Table 2:** Bending length and flexural rigidity of untreated and treated hemp fabrics.

Sample	Bending Length (cm)		Flexural Rigidity (mg·cm)	
	Warp	Weft	Warp	Weft
Hemp	1.86 ± 0.28	1.78 ± 0.26	17.12 ± 3.25	13.97 ± 2.91
Hemp + ZnO	2.11 ± 0.30	2.95 ± 0.26	29.01 ± 2.81	40.43 ± 3.04
Hemp + 2%Soy + ZnO	2.76 ± 0.23	2.89 ± 0.29	59.28 ± 3.34	72.39 ± 2.87
Hemp + 2%Soy + ZnO + Binder	3.67 ± 0.26	4.16 ± 0.23	151.75 ± 4.21	215.68 ± 3.61

**Table 3:** Tensile strength and elongation at break of untreated and ZnO-functionalized hemp fabrics.

Sample	Tensile Strength (N)		% Elongation at Break	
	Warp	Weft	Warp	Weft
Hemp	142.95 ± 14.98	149.65 ± 32.36	26.55 ± 2.54	23.57 ± 3.49
Hemp + ZnO	111.42 ± 21.63	130.08 ± 22.36	21.40 ± 1.19	25.15 ± 3.45
Hemp + 2%Soy + ZnO	130.08 ± 16.62	143.19 ± 19.40	22.05 ± 1.90	24.76 ± 4.46
Hemp + 2%Soy + ZnO + Binder	94.87 ± 11.01	113.58 ± 25.94	17.23 ± 4.52	24.82 ± 2.80

**Table 4:** UPF, UV protection performance and thickness of hemp fabrics under different finishing.

Sample	UPF	UVA (%)	UVB (%)	Thickness (mm)
Hemp	21.10 ± 3.44	91.40 ± 2.91	94.50 ± 1.64	0.66 ± 0.01
Hemp + ZnO	32.50 ± 2.21	95.60 ± 2.63	96.60 ± 1.13	0.76 ± 0.01
Hemp + 2%Soy + ZnO	50+	98.40 ± 1.21	98.50 ± 1.84	0.76 ± 0.02
Hemp + 2%Soy + ZnO + Binder	50+	99.30 ± 0.50	99.40 ± 0.52	0.77 ± 0.01

**Table 5:** Zone of inhibition values (mm) against *E. coli* for untreated hemp, ZnO-coated, and soy-assisted ZnO-functionalized hemp before and after washing.

Sample	Zone of Inhibition (mm)	Agar Diffusion Image
Hemp	0.0	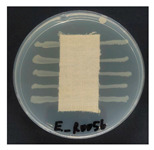
Before wash		
Hemp + ZnO	5.7	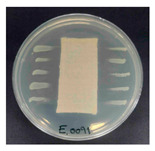
Hemp + 2%Soy + ZnO	6.4	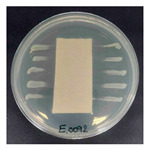
After wash		
Hemp + ZnO	3.2	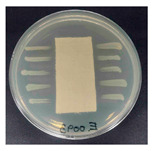
Hemp + 2%Soy + ZnO	3.8	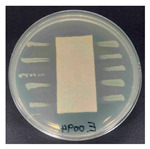

**Table 6:** Zone of inhibition values (mm) against *S. aureus* for untreated hemp, ZnO-coated, and soy-assisted ZnO-functionalized hemp before and after washing.

Sample	Zone of Inhibition (mm)	Agar Diffusion Image
Hemp	0.0	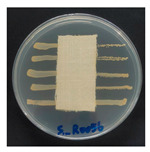
Before wash		
Hemp + ZnO	5.6	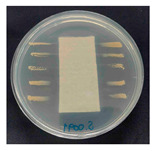
Hemp + 2%Soy + ZnO	5.3	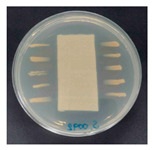
After wash		
Hemp + ZnO	2.8	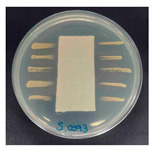
Hemp + 2%Soy + ZnO	2.7	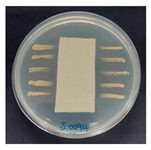

**Table 7:** Antibacterial activity of finished textile materials assessed by AATCC TM 100 against *S. aureus* for untreated hemp, ZnO-coated, and soy-assisted ZnO-functionalized hemp before washing.

Sample	% Reduction	Agar Diffusion Image
Hemp	0.00	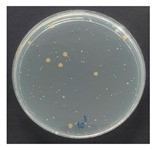
Before wash		
Hemp + ZnO	>99.99	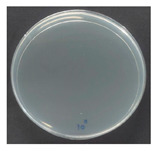
Hemp + 2%Soy + ZnO	>99.99	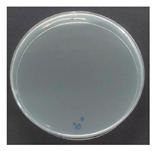

## Data Availability

The original contributions presented in this study are included in the article. Further inquiries can be directed to the corresponding author.
